# Anticipating and strategizing to address potential bottlenecks during clinical research projects in sub-Saharan Africa: a case for adapting approaches and tools used in the entrepreneurial and development sectors

**DOI:** 10.1186/s13063-023-07897-7

**Published:** 2024-02-03

**Authors:** Yauba Saidu, Sangwe Clovis Nchinjoh, Amani Adidja, Andreas Frambo Besong, Palmer Masumbe Netongo, Paul Ndom, Marya Liebermann

**Affiliations:** 1 Clinton Health Access Initiative Inc, PO Box 2664, Yaoundé, Cameroon; 2 Institute for Global Health, University of Siena, Siena, Italy; 3 Faculté de Médecine Et de Sciences Biomédicales de L, Université de Yaoundé I (FMSB-UYI), Yaoundé, Cameroon; 4 Faculty of Life Sciences, University of Yaoundé 1, Yaoundé, Cameroon; 5 Department of Chemistry and Biochemistry, University of Notre Dame, Notre Dame, South Bend, IN USA

## Abstract

Many research funders have invested billions of US dollars in building research capacity in sub-Saharan Africa (SSA). Despite these colossal investments, many well-intentioned and designed clinical research projects have either failed to kick off or ended abruptly. Although obstacles to clinical research in SSA are well known, there is limited information on frameworks and tools that can be used to anticipate and avert these systemic bottlenecks, particularly those related to socio-politics. In this paper, we leveraged lessons from entrepreneurs and development experts in harsh and uncertain business environments to develop a framework for anticipating and addressing potential bottlenecks to clinical research in SSA. More so, to illustrate and build a case for this framework, we shared our experience in supporting clinicians and regulators to adopt a point-of-use care tool, the “chemoPAD,” to screen for the quality of anticancer medications rapidly and systematically in Cameroon despite resistance from some stakeholders. The critical steps in this framework involve identifying stakeholders, categorizing them based on their potential reactions to the study (adversary, supporters, and indifferents), and developing critical strategies to engage or deal with each stakeholder’s reactions, starting with adversaries. This approach may be useful in complex research projects, especially clinical trials, which often involve many stakeholders with different interests and perceptions.

## Introduction

Clinical research, especially clinical trials, plays an essential role in public health, including the development of effective diagnostics, drugs, vaccines, clinical methods, and even behavioral change models that can help save lives and improve well-being in sub-Saharan Africa (SSA). Given this promise, many funders, especially foreign research-oriented institutions, have invested billions of United States (US) dollars in building research capacity in SSA to enable institutions in this part of the world to execute research projects in accordance with internationally accepted standards and principles [1, 2]. Most of these efforts have been geared towards upgrading research infrastructures, equipping institutions with state-of-the-art equipment, and training investigators and their teams on designing protocols and building systems to run clinical research projects [1]. Efforts have also been tailored towards building the capacity of ethics and regulatory bodies to review research protocols and provide adequate oversight during the implementation of research projects [1].

Despite these colossal investments, many well-intentioned and designed clinical research projects have either failed to kick off or ended abruptly. For instance, in early 2022, over 100 multimillion-dollar research projects funded by major donors, including the Bill & Melinda Gates Foundation (BMGF), the Swedish Development Corporation Agency (SIDA), Welcome Trust, the Department of International Development (DFID), and the New Partnership for Africa's Development (NEPAD), were put on hold at the African Academy of Science [3]. Similarly, during a large Ebola outbreak in West Africa in 2014, the Gambian government stopped the Gambian Medical Research Council (MRC, the Gambia) from conducting an Ebola vaccine trial—a £2.8 M investment from Welcome Trust, MRC, and the UK government [4]. Likewise, in 2005, the government of Cameroon abruptly stopped the tenofovir study for HIV prevention—a US $6.5 M investment from BMGF [5]. In 2009, Pfizer was requested to pay US $75 M for a trial it conducted in Nigeria in 1996, which the government considered to be unethical and caused the death of many children [6]. These examples highlight the extremely challenging environments that donors and researchers in SSA encounter in their quest to address some of the most pressing health needs in the continent.

Interestingly, outright political kickback from governments is only one of the challenges donors and researchers face in this part of the world. Other challenges extend beyond socio-politics and include bottlenecks like unpredictable governance, unpredictable supply chain and logistic systems, unclear ethical and regulatory requirements, pathways and timelines, poor infrastructure, and limited or unreliable access to enabling technologies (IT, diagnostics, treatment technologies, banking services, among others), official inertia, lack of support from research communities, high illiteracy levels, bureaucratic foot-dragging, and outright corruption among others. Many of these obstacles, particularly those related to ethics and the smooth conduct of clinical trials, have been extensively studied and solutions proposed [7–13].

**Fig. 1:**
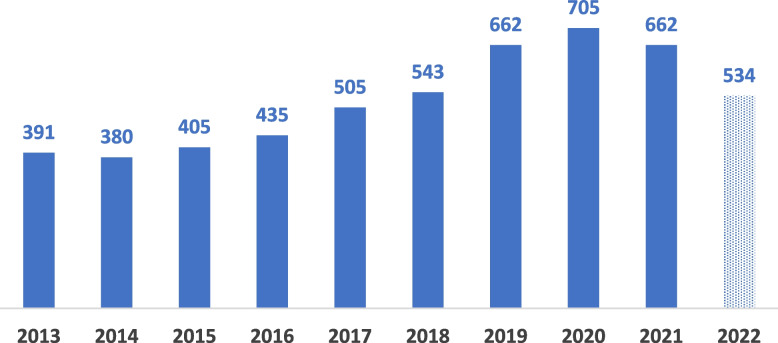
The number of trials conducted in Africa during the past ten years. This column chart shows a gradual increase in clinical research conducted in Africa, from 391 in 2013 to 705 in 2020. However, from 2020 a significant drop to 534 in 2022 was reported—probably due to the impact of the COVID-19 pandemic

However, obstacles to creating an enabling environment for clinical research, including systems and tools that can enable funders and researchers to easily navigate complex environments and detect and address potential bottlenecks before project initiation, seem to have received limited attention from the scholarly community. As a result, it is not surprising to see that many well-designed and well-intentioned clinical research projects fail to achieve their goals or end uninformatively. For example, according to the World Health Organization’s International Clinical Trials Registry Platform (ICTRP), which pulls all major trial registries, a total of 5222 clinical trials were conducted in SSA between January 2013 and November 2022 (Fig. 1). However, several commentators felt that many of these trials, particularly those related to Covid-19, ended uninformatively, leading to a waste of time and resources. In addition, the outcomes of many of these trials are unknown, as many principal investigators rarely update trial registries at the end of their trials due to selective bias in reporting trial outcomes [14, 15]. Because of this limitation, it is difficult to tell how many of these trials failed to kick off or ended abruptly—an outcome that may be difficult to rule out given some of the examples cited above.

One overarching question is how this phenomenon of clinical research projects failing to kick off or ending abruptly in the region can be prevented. A potential solution to this question is to leverage and apply lessons from failures in international development or entrepreneurial sectors. In the business world, reports suggest that 22% of startups fail within the first year and 50% within the first 5 years, and failure rates tend to increase over time [16]. Indeed, the World Bank has previously reported a failure rate of over 50% of its development projects in Africa, and the rate is similar for many donors [17]. This high failure rate has led some scholars to describe SSA as “a graveyard” for several development projects that have left the beneficiaries worst off [18]. These failures have taught several entrepreneurs and development experts to devise strategies to successfully mitigate adverse outcomes during project implementation. These lessons can also be translated into the clinical research sphere. In this paper, we present how some of these lessons can be leveraged to improve the likelihood of success of clinical research projects in SSA. We also share a case study in which we applied the approach to preempt the project from failing to kick off despite outright opposition from some stakeholders.

### Overview of the case study—improving the quality of anticancer medications in sub-Saharan Africa

In 2020, about 19 million newly diagnosed cancer cases and over 10 million related deaths (53%) were reported globally [19]. The proportion of deaths was over 10 points higher (66%) in SSA, a figure which is projected to double by 2030 [20]. Similarly, the survival rate of cancer patients in SSA is twofold lower compared to high-income settings. For example, the 5-year survival rate for women with breast cancer in the USA is estimated at 90%—higher than survival rates in SSA countries such as Uganda (46%) and the Gambia (12%) [21]. This considerably lower survival rate seemingly results from a combination of reasons, including late diagnosis, lack of access to treatment, and high use of substandard or falsified (SF) chemotherapy drugs.

Studies have shown that treatment with chemotherapy medications containing less than 65% of the recommended dose level resulted in outcomes similar to cases without treatment [22]. Therefore, assuring the quality of chemotherapy drugs is critical to ensure they yield the desired treatment outcomes. However, many countries in SSA, including Cameroon, do not have robust systems and tools to ascertain the quality of drugs before issuing marketing authorization. The countries also lack robust systems to enable them to conduct postmarket surveillance (PMS) activities to detect the circulation of SF medications. The lack of inexpensive, portable, and reliable technology for detecting SF chemotherapy drugs at the point of care has been a driving force behind the limited PMS on chemotherapy drugs in SSA. This problem is particularly worrying given that these medications make an attractive target for falsification due to their high prices. These high prices, and the resulting profits, are often attractive to diverse and powerful stakeholders, who have established parallel supply chains to enhance the distribution and sales of SF products. As a result, any research endeavor to solve this pervasive problem may likely encounter serious kickbacks from the distributors of SF chemotherapies.

Our project, “adopting a point-of-use card, the ChemoPAD, to assess the quality of chemotherapy products in Cameroon,” was primarily designed to provide clinicians and regulators with a point-of-care tool that they can use to screen for the quality of anticancer medications in the country. Like many well-intentioned projects, this project funded by the United States National Institute of Health (NIH) could have failed to kick off because its approval could be blocked by some powerful stakeholders. Given that naivete to political realities has compromised, delayed, or destroyed many well-intentioned projects in Africa, it was critical for us to develop and leverage a sociopolitical strategy to initiate our project. The goal of the strategy was to preempt adverse effects of inevitable socio-politics on our project. In this paper, we described the approach we used to mitigate the risk of our project failing to kick off.

### The proposed approach for anticipating and dealing with potential bottlenecks during the implementation of a research project

Each clinical research project presents unique sociopolitical challenges as it often involves multiple stakeholders with diverse interests. As a result, naivety to political realities may compromise, delay, or even ruin well-designed clinical projects. To preempt this adverse outcome, establishing a sociopolitical strategy before initiating a clinical research project may be mission-critical for its success. Over the years, we have learned that developing such a strategy involves four critical steps, including the following: (1) *identification of all stakeholders* whom the project may impact in one way or another, (2) *categorizing the identified stakeholders* based on how they may impact the project, and (3) and (4) *developing a roadmap* that outlines a comprehensive strategy on how to engage each identified stakeholder. The section below provides a summary of each step.

#### Step 1: Identification of stakeholders

The first step towards developing a robust strategy to deal with the inevitable socio-politics during project implementation is conducting an extensive mapping and characterization of all the stakeholders. This involves listing all the people, organizations, institutions, or departments that may be impacted by the project. This step should be followed by a pre-characterization of each stakeholder in terms of how each of them will be impacted by the project, which might either be positive (projected positive impact) or negative (projected negative impact). It is also important to brainstorm and document when the projected impact might occur — in other words, whether it would occur in the short term, medium term, or long term. Once this has been documented, the next step should focus on brainstorming on each party’s possible reaction (either positive or negative) to the project. It is worth noting that some stakeholders might be impacted negatively or positively; in such a case, negative impacts most often occur in the short term. The final step in this identification process is to capture the outcomes of the brainstorming exercises in a table of stakeholders (TOS) (Table 1). Ideally, this TOS should be updated as progress is being made because new stakeholders may emerge, and the perceived impact of the research process and other project dynamics may change over time. The TOS should be discussed with the project’s advisory board members, who are generally more experienced and will provide valuable insights that can guide the strategies to be adopted and deployed in the event of any adverse impact.

**Table 1:** Sample table of stakeholders

	**Impact (short, medium, long term)**	
**Stakeholder**	**Major negative**	**Major positive**
Funder		
Relevant ministry		
Regional boards/delegations		
Religious leaders		
Local leaders (village elders)		
Political leaders		
Administrative authorities		
Community advisory board		
Healthcare workers		
Policy makers		
Ethics committee		
Regulators		
Social media influencers		
Key opinion leaders		
Participants and families		
Suppliers/service providers		
Customs		
Etc		

#### Step 2: categorization of identified stakeholders

**Fig. 2:**
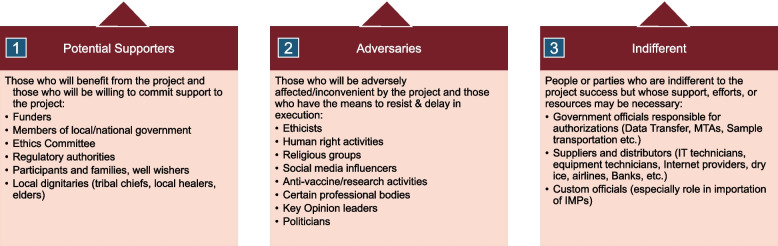
Categorization of stakeholders. This figure shows a sample grouping of stakeholders into potential supporters, adversaries, and indifferents

The TOS might be long as it will present different pieces of information for each identified stakeholder, including potential impacts and perceived reactions. Still, to effectively develop an engagement strategy, it is essential to categorize the identified stakeholders into one of three groups, as shown in Fig. 2. As illustrated, these categories include potential supporters or allies, potential adversaries, or opponents, and indifferents. *Potential supporters* are those who will benefit from the project, such as participants and their families, as well as those who would be willing to commit support to the project, such as funders or local ethics or regulatory bodies who understand the value of the research project. On the other hand, *adversaries* are those who may be adversely affected or inconvenient by the project, such as certain religious groups or societal bodies (e.g., anti-vaccine lobbyists), including those who have the means to resist or delay the project’s execution, such as politicians and some key opinion leaders (KOL). It is advisable to identify potential adversaries as early as possible and establish a strategy on how to deal with their misgivings and reactions. The last category, *indifferents*, is stakeholders who might be indifferent to the research project’s success but whose support, efforts, or resources may be necessary for the project’s success. These may include certain government officials whose authorizations are needed to import or export certain research materials and supplies. The list also extends to suppliers or distributors such as banking institutions, Internet providers, dry ice manufacturers, airline companies, customs, or biomedical engineers. Often, the support or collaboration of these *indifferents* may be critical to the operations of the research project, but these stakeholders may consider the research project as not worthy of their time or beneficial for them financially. For instance, a supplier dealing with dry ice may not see any financial incentive to supply 10 kg of dry ice to a researcher who needs to ship biological samples in negative temperatures to Europe or the USA for analysis. Similarly, an airline company may refuse to transport biological samples if they are aware that the samples were collected from patients with hemorrhagic fever. Still, customs can seize and destroy clinical research materials that have not received the necessary authorization for importation.

A critical element to monitor is the change of stakeholders over time, particularly for projects with a long duration as new stakeholders may appear, while initial stakeholders may exit the scene over time. Initially, most of these stakeholders may be indifferent or inactive to the project; however, this perspective may change over time. For this reason, it would be important to anticipate the potential response from each of the stakeholders and when this might occur during the project, particularly during the early phase of the research. Typical uncertainties often encountered during this phase are the initial responses from stakeholders. In the face of these uncertainties, using a “fail-safe” method may minimize potential adverse consequences to the project [23]. For instance, planning to deal with initial opposition when the researcher is unsure about the type of reaction he will get from his adversaries, supporters, or indifferents may spare him disillusions from lack of support or frustrations from unforeseen opposition. Similarly, the researcher may also plan to deal with a rapid response from an adversary when he is unsure whether the potential response will be rapid or delayed so he is not caught slumbering. Also, the researcher can plan to deal with a delayed response from supporters and indifferents, so he is not frustrated if he experiences delays or lack of support from these stakeholders. And finally, given the numerous stakeholders, it would be important for the researcher to establish the degree of power and influence that each stakeholder may have to propel or impede his project.

#### Step 3: develop a strategy for dealing with each stakeholder

**Fig. 3:**
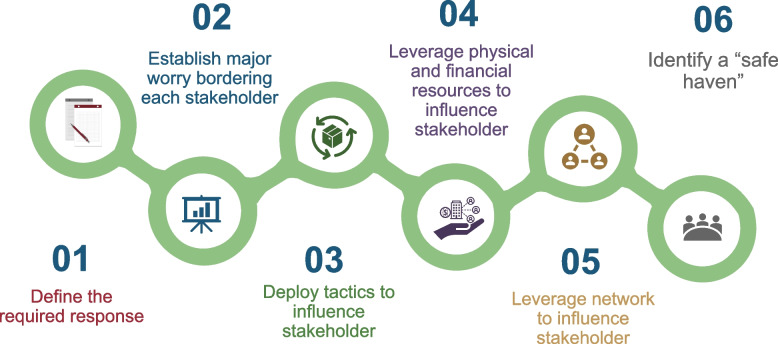
Steps for developing a strategy for dealing with identified stakeholders. This gives an illustrative step-wise approach to developing strategies to manage stakeholders based on their position relative to the research

Not having a plan to deal with the reactions from the different stakeholders may be a pretty good sign that the project might run into problems, which may lead to a significant waste of effort, money, and time. As a result, developing a strategy for mobilizing supporters, managing adversaries, and energizing indifferents may pay off. The strategy should be robust enough to deal with each stakeholder’s reactions as captured in the table of stakeholders. Figure 3 summarizes the steps that should be followed to develop such a strategy.

The first step involves defining the response the researcher will need from a given stakeholder. This response should center around three core elements, namely: (1) getting potential adversaries to commit not to antagonize the project, (2) galvanizing support from indifferents, and (3) getting supporters to pledge their support to the project or to grant the researcher access to their networks, which he can subsequently deploy to influence another stakeholder.

The second step consists of establishing major issues bothering a given stakeholder. This involves collecting intelligence on the major preoccupations of each stakeholder and information on the broader context of the research setting to understand the issues shaping the actions and behaviors of each critical stakeholder. This exercise may also help unveil some aspects of the project to which the stakeholders may be most sensitive and reactive to, as well as help uncover the basis for potential stakeholder antagonism, support, or indifference to the project. Understanding these issues may enable the researcher to deploy tactics that may act in his or her favor.

The third step consists of leveraging insights from the intelligence gathered in step 2 to deploy tactics to influence the different stakeholders. This may include helping supporters resolve their major concerns to leverage their support, including accessing their allies where needed. It may also involve educating adversaries about the importance of the research project or deploying tools and knowledge to help them resolve issues pressing to them or using allies to mitigate opposition from the adversaries. It may also involve energizing indifferents with resources, tools, and knowledge to improve their position.

The fourth step may consist of deploying resources (including funds, properties, equipment, or materials) that the researcher possesses, where necessary, to recompense supporters and indifferents for their assistance or adversaries to stop their antagonism.

The fifth step involves leveraging and deploying the available network to influence the different target stakeholders. Here, the researcher can use his supporters or their allies to galvanize support from indifferents or counteract adversaries. If the researcher is unable to deal with opposition from potential adversaries, he or she should leverage support from his allies or supporters who can protect him or her from retaliatory actions from the opponents.

The sixth step in the strategy development face is to identify a location, pathway, or “safe haven” where the research project can be successfully implemented without outright opposition from opponents. This consists of identifying a “protected niche” upfront where the project can be implemented without intense opposition. This “protected niche” can be a new setting or location that the adversary has no influence over. It may also involve postponing or delaying the start of the project or collaborating with organizations or institutions that the opponent has no influence over. For large and potentially controversial projects, identifying a “godfather,” “godmother,” or an “organization” that can heat-shield the project and guide it through cultural, ethnic, religious, political, or bureaucratic environments may be mission critical.

#### Step 4: develop a tactic table for managing each stakeholder

After developing the strategy, the next step should focus on developing a tactic table for each stakeholder, beginning with the most prominent adversaries. These are the stakeholders who can impede or retard the initiation of the project either through open or reflexive resistance. Failure to properly manage adversaries at this stage may prevent the project from kicking off. The tactic table for each stakeholder should clearly map out the anticipated outcomes. For adversaries, the tactic should aim at blocking, avoiding, or limiting their actions, while for allies or supporters, the tactic should aim at securing their support or the support of their allies. And for indifferents, the tactic should aim to persuade them to support the project.

In our case study, “Adapting a point of use card, the ChemoPAD, for the assessment of the quality of chemotherapy products in Cameroon,” we leveraged this process to develop tactic tables for each category of the identified stakeholders. These stakeholders included:

- *Potential adversaries*: Distributors of SF chemo drugs (distributors, pharmacies, medical delegates, and a network of prescribers)
- *Supporters/allies*: A leading teaching hospital (safe heaven) for patients and caregivers, Ministry of Public Health, and clinicians
- *Indifferent*: Drug regulators and ethics committees

Tables 2, 3, and 4 are the tactic tables we developed for each stakeholder.

**Table 2:** Tactic table for primary adversary (dealers in SF chemotherapies)

**Expected outcome**: Avoid adversaries blocking ethical and regulatory approvals for testing chemoPAD in cancer treatment centers as well as conducting the research in Cameroon	
**Major issue occupying stakeholders**: Approval for the use of chemoPAD to screen for quality of anticancer drugs may lead to a disruption of a long-standing & viable business	
**Tactic**	**Response**
Deploy strategies to solve adversary’s problem to garner influence for horse trading	No
Deploy strategies to aggravate the adversary’s position	No
Reward adversary for cessation of opposition	No
Leverage support from allies or ally’s allies to mitigate antagonism or protect project from adversary	No
Identify a protected niche where adversary has no influence	University teaching hospitals and oncologies are interested in seeing desired outcomes in patients receiving chemotherapy

**Table 3:** Tactic table for cancer treatment center at university teaching hospitals

**Expected outcome**: Approval for the conduct of the chemoPAD study	
**Tactic**	**Response**
Deploy knowledge and skills of the issues facing quality anticancer therapy in Africa	The study team has deep experience and knowledge of the state of the problem of sub-standard and falsified drugs in Africa, and this can be deployed to help physicians understand why they do not see a clinical response following chemotherapy sessions
Deploy physical and financial resources to influence stakeholder	No—the study team has no such resources
Deploy network and or ally’s network to influence ally?	Leverage our connections with cancer treatment centers in Africa and the USA to share their experiences with hospital administration and clinical staff on they solved this problem

**Table 4:** Tactic table for the indifferent drug regulators

**Expected outcome**: Approval for the conduct of the chemoPAD study and use of study results to guide the registration of chemotherapy drugs in the country	
**Major current issue occupying stakeholder attention**: The regulatory authority does not have the tools and capabilities to screen for the quality of anticancer drugs before authorizing their use in the market or tools to check for the circulation of SF drugs in the market	
**Tactic**	**Response**
Deploy capabilities to solve indifferent problems in order to their secure support	• Train regulators on screening for the quality of anticancer drugs • Arranged for the laboratory arm of the regulatory agency to go for an exchange visit to a drug testing laboratory in the USA for capacity building
Deploy resources to galvanize support from indifferent	• The study team does not have such resources
Leverage allies or ally’s allies in our network to mobilize indifferents	• Organize a meeting between the lead oncologist and regulators so that the oncologist can share their experiences with the regulators

The above approach was used to successfully obtain all the necessary approvals for the study, despite some delays and numerous back and forth with the ethics committee that some adversaries might have likely inspired.

Using the above approach can enable researchers to successfully anticipate and mitigate obstacles that could prevent their projects from kicking off or ending abruptly. A tool that tries to summarize this approach is currently being developed by the “Design, Analyze and Communicate” (DAC) arm of the Bill & Melinda Gates Foundation (9). Although designed with a specific focus on clinical trials, this tool can help researchers successfully mitigate several challenges that they may encounter during the implementation of their projects. Essentially, the tool is a fillable tool with a set of standard prompting questions and non-required questions developed to provide the minimum requirement for effective communication while at the same time allowing for remodeling to suit the research context.

## Conclusion

Clinical research, especially clinical trials, will continue to be important in disease prevention and control in sub-Saharan Africa. However, researchers in this part of the world face an extremely punishing environment that most often prevents research projects from kicking off or causing them to end abruptly. As a result, researchers would need an arsenal to help them navigate uncertain environments to preempt their projects from being compromised, delayed, or destroyed by the prevailing socio-politics in these settings. In this paper, we shared our experience, which is based on lessons from the development and entrepreneurial sectors, to successfully anticipate and mitigate potential bottlenecks that might have prevented our research project from kicking off. We used a systematic approach to first identify all potential stakeholders who might be impacted by the project, then categorized them based on their potential reactions (adversary, supporters, and indifferents), and established a strategy on how to engage or deal with their concerns. Such an approach may be replicated, with success, in many settings across sub-Saharan Africa.

## Data Availability

Not applicable.
